# Molecular mechanisms underlying phytochrome-controlled morphogenesis in plants

**DOI:** 10.1038/s41467-019-13045-0

**Published:** 2019-11-19

**Authors:** Martina Legris, Yetkin Çaka Ince, Christian Fankhauser

**Affiliations:** Center for Integrative Genomics, Faculty of Biology and Medicine, University of Lausanne, CH-1015 Lausanne, Switzerland

**Keywords:** Light responses, Plant signalling

## Abstract

Phytochromes are bilin-binding photosensory receptors which control development over a broad range of environmental conditions and throughout the whole plant life cycle. Light-induced conformational changes enable phytochromes to interact with signaling partners, in particular transcription factors or proteins that regulate them, resulting in large-scale transcriptional reprograming. Phytochromes also regulate promoter usage, mRNA splicing and translation through less defined routes. In this review we summarize our current understanding of plant phytochrome signaling, emphasizing recent work performed in Arabidopsis. We compare and contrast phytochrome responses and signaling mechanisms among land plants and highlight open questions in phytochrome research.

## Abstract (web summary)

Perception of red and far-red light by photosensory phytochromes regulates plant development and adaptive responses to light. Here, Legris et al. review current models of phytochrome function connecting light-induced conformational changes to physiological outputs and highlight open questions for future research.

## Introduction

**Fig. 1:**
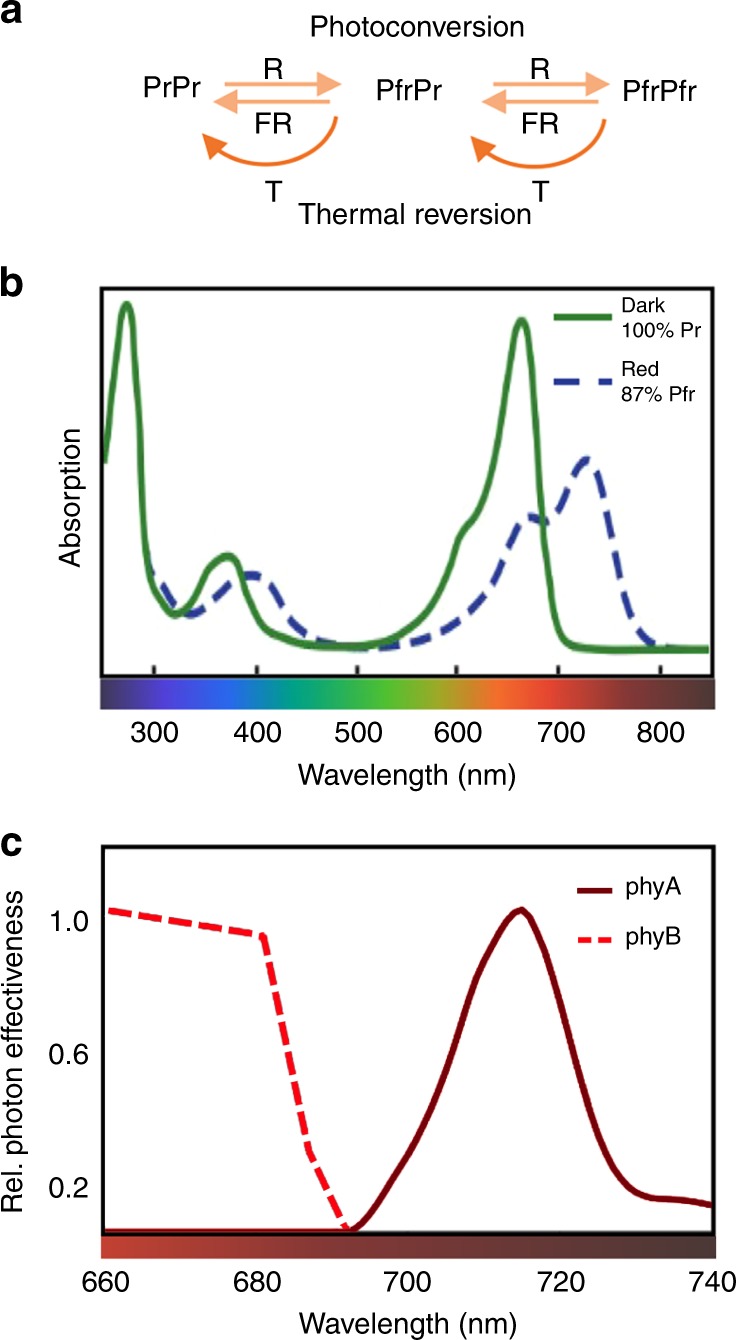
Control of phytochrome activity. **a** Factors controlling phytochrome activity. Phytochromes exist in two conformations, Pr and Pfr, the latter being the active form. They exist as dimers so three species can be found. Each monomer can be activated by red light (R) and inactivated by far-red light (FR) or by thermal reversion, a process that depends on temperature (*T*). At least in the case of phyB, Pfr in heterodimers reverts much faster than that in homodimers, allowing phyB to perceive temperature both during the day and during the night. **b** Plant phytochrome absorption spectra of the Pr and Pfr conformations. In dark-adapted seedlings phytochromes are in the Pr form. Upon a saturating R pulse, due to overlapping absorption spectra of Pr and Pfr, only 87% of Pfr is achieved. **c** Action spectra for phyA and phyB in the control of hypocotyl elongation. Data from Klose et al.^3^. Fluence rate response curves are measured at different wavelengths and fluence rate that leads to 40% inhibition compared with dark control is determined. In order to specifically determine action spectra for phyA and phyB, for phyB the curve was performed with phyB-GFP/*phyAphyB* seedlings, and for phyA using *phyB-5* seedlings. Values are relative to the response obtained at the most efficient wavelength in each case

Phytochromes are present in bacteria, cyanobacteria, fungi, algae, and land plants, and while in all cases they can perceive light, their photochemical properties vary largely among phyla^1,2^. In this review we will focus on land plant phytochromes, and in particular on Arabidopsis, for which we have a better understanding of the underlying molecular mechanisms. In land plants, phytochromes are red and far-red light receptors that exist in two forms. They are synthesized in the inactive Pr state, which upon light absorption converts to the active Pfr conformation. Pfr is inactivated upon far-red (FR) light absorption or through thermal relaxation, which depends on temperature, a process known as dark or thermal reversion. Phytochromes act as dimers, resulting in three possible phytochrome species: Pr–Pr, Pfr–Pr, and Pfr–Pfr^3^ (Fig. 1a). Pr and Pfr have different absorption maxima, but due to overlapping spectra both conformers are always present in the light while only prolonged darkness returns all phytochrome to Pr (Fig. 1b)^3^. Given that phytochrome responses depend on the proportion of Pfr conformers, signaling is influenced by a combination of light quantity, color, and temperature^3–5^.

Upon perception of inductive wavelengths, activated phytochromes together with blue (phototropins, cryptochromes, and Zeitlupes) and UV light receptors (UVR8) control plant physiology and development^6,7^. Once the seed is imbibed, active phytochromes promote germination^6^. When a seedling grows in the soil it adopts an etiolated morphology characterized by fast-growing hypocotyls and closed apical hook, which maximizes the chance to reach the surface. Once it reaches the light, activated phytochromes promote de-etiolation: hypocotyl growth slows down, the apical hook opens, cotyledons expand, and chloroplasts develop^6^. This initial response to light occurs even in poor light conditions as encountered under deep shade where blue and red light (R), which are essential for photosynthesis, are scarce^6^. Unfiltered sunlight has approximately equal amounts of R and FR resulting in a ratio of R to FR (R/FR) slightly above 1 and high phytochrome activity^8^. However, in environments with high plant density the R/FR drops since green tissues absorb mainly R and blue light and transmit or reflect FR. This results in reduced phytochrome activity triggering the shade-avoidance response in green seedlings. Stem and petiole elongation are promoted, leaves change their position and anatomy, root architecture is altered, while senescence is promoted^8–10^. In these conditions, plants allocate more resources to growing aerial parts^11^ and change their metabolism^12^. Increasing temperature promotes similar architectural changes as shade^13^ and a subset of these temperature responses depend on phytochromes^4,5,14^. Moreover, phytochromes are important to entrain the circadian clock and for the perception of the photoperiod^6,15,16^. These signals in addition to temperature and light quality control flowering time^6,17–19^.

In this review we summarize the current knowledge on early phytochrome signaling mechanisms in Arabidopsis with an emphasis on de-etiolation and shade avoidance, the photomorphogenic processes for which most information is available. Other phytochrome-controlled developmental processes are discussed in the context of long-distance signaling. Comparisons between Arabidopsis and other species for which molecular or genetic data are available is presented in a separate chapter. Due to space constraints we could not systematically refer to the older primary literature but we suggest more focused review articles where historical perspectives can be found.

## The phytochrome family in Arabidopsis

Most land plants possess several phytochromes, and in most angiosperms three groups can be identified: phytochrome A (phyA), phyB, and phyC^2,20^. Arabidopsis has one member in the phyA and phyC groups while the phyB class is composed of phyB, phyD, and phyE^6,14,21,22^ (Table 1). Each phytochrome has different roles and their relative contributions vary depending on the environmental conditions and developmental stage of the plant (Table 1)^14,21–25^. Angiosperm phytochromes are further classified into two categories according to their capacity to trigger responses to specific light signals. Type I, represented by phyA in Arabidopsis, are light labile and allow germination and de-etiolation when light is scarce (Very Low Fluence Response or VLFR) or when the R/FR is very low (FR-High Irradiance Response, FR-HIR)^6^. Such conditions are encountered under a thin layer of soil and in deep shade. Type II phytochromes (phyB–phyE in Arabidopsis) are light stable but require a substantial fraction of Pfr to promote signaling. Therefore, they are active in more open environments over a wide range of irradiances where the R/FR is relatively high (low fluence response, LFR)^6^. Hence, despite very similar absorption spectra type I and type II phytochromes have different action spectra^3^ (Fig. 1b, c, see Box 1). The contribution of each phytochrome to light-regulated plant development can be explained by their different expression levels, protein structure, protein stability, and photochemical properties, as well as the mechanisms underlying their nuclear internalization^3,6,14,24,26,27^ (see Box 1 and Table 1 for details). In addition, each phytochrome has a different contribution to temperature responses with phyB having a major role. Active phyB is a Pfr–Pfr homodimer and the rate of thermal reversion of the Pfr–Pr heterodimer is much faster than that of the active Pfr–Pfr homodimer^3^ (Fig. 1a). The occurrence of this fast reversion allows phyB to perceive temperature not only during the night but also in the light^4,5^. According to modeling approaches temperature-enhanced phyB inactivation is expected to significantly antagonize light activation during cloudy days or in shaded environments^28^. In contrast, phyA is not expected to contribute to such temperature responses, as its thermal reversion rates are much slower than those of phyB at least in some accessions^29^. In addition to their individual roles, type II phytochromes can form heterodimers^24,30^. Importantly, in Arabidopsis phyC mostly works as a heterodimer with other phytochromes and the phyB–phyC heterodimer is particularly important to inhibit flowering in non-inductive photoperiods (Table 1)^14,23^. Despite their important roles throughout the plant life cycle and the strong phenotypes of higher order phytochrome mutants, Arabidopsis can complete its life cycle in the lab without phytochromes^14,31,32^.

**Table 1:** Functional diversification of the phytochrome family in *Arabidopsis*

Phytochrome	phyA	phyB	phyC	phyD	phyE
Type (light responses)	I	II	II	II	II
Class (phylogeny)	A	B	C	B	B
Level in etiolated seedlings^26^	85	10	2	1,5	1,5
Level in the light^26^	5	40	15	15	25
Germination^14,25^	WLc. VLFR, HIR	WLc LFR	Can't promote on its own but can interact synergistically with other phytochromes	WLc Major role in RLc, but can't respond to Rp Synergy with phyA in FR	Poor inducer on its own but can interact synergistically with other phytochromes
De-etiolation	VLFR, HIR	LFR	LFR Not sufficient	LFR Not always sufficient	LFR Not always sufficient
Shade avoidance	Negative regulation	Dominant	No function on its own	Small effect, in combination with phyB^22^	Small effect, in combination with phyB^21^
Flowering	Weak repressor. Antagonizes phyD and phyE Can confer photoperiod sensitivity on its own	Strong repressor. Together with phyC necessary for flowering in response to photoperiod	Repressor in SD^23^ Together with phyB necessary for photoperiodic flowering	Weak repressor	Strong repressor Temperature dependent
Dimers^30^ ^a^	Homodimers	Both	Heterodimers	Both	Both^b^
Nuclear internalization^57^	Mediated by FHY1 and FHL	Independent of FHY1 and FHL. Possibly mediated by PIFs	Dependent on dimerization with phyB (or phyD)	Independent of FHY1 and FHL. Not light regulated in the absence of phyB	Independent of FHY1 and FHL

*WL* white light, *R* red light, *FR* far red light, *c* continuous, *p* pulse, *SD* short days

^a^Sanchez Lamas et al.^14^ could detect all possible dimers with the exception of phyC/phyC using BiFC

^b^Although they were not detected in Co-IP^30^, they were found in native western blots^24^, and phyE alone can repress flowering efficiently^14^

Box 1 Differences between phyA and phyB PhyA and phyB are the most abundant phytochromes in Arabidopsis and the most important for photomorphogenesis. While their absorption spectra are nearly identical, they can control responses to different environmental light cues. The responses triggered by phyB are easily explained because the phyB action spectrum matches the photoreceptor absorption spectrum (Fig. 1). In other words, light that promotes the formation of the active PfrB triggers the response while light that returns the active conformer back to its inactive PrB state inhibits the response. These responses are known as the low fluence response (LFR), exemplified by the classic lettuce seed germination experiment showing red light induction and far-red reversibility. While phyA can also act in the LFR mode, it is best known for two other modes of action: the response to broad spectrum very low amounts of light and to high irradiances of FR light (VLFR, FR-HIR). These two modes of action have in common that they initiate phyA responses in the presence of very low levels of active PfrA and are irreversible. Irreversibility is a consequence of the overlapping absorption spectra of Pr and Pfr implying that any wavelength (even FR < 750nm) leads to a small fraction of PfrA.

The typical modes of phyB and phyA action differ in four major ways: (1) Reversibility of the response. (2) The action spectrum, which is significantly shifted towards FR in phyA no longer matching the PrA absorption spectrum, while for phyB action and absorption spectra are similar. (3) A substantial fraction of phyB needs to be Pfr to trigger a response while for phyA a much smaller fraction is sufficient. (4) PfrB has to be present for some time to initiate a response while for phyA even in conditions leading to rapid Pr–Pfr–Pr cycling the response is elicited.

The following differences between phyA and phyB contribute to our understanding of how these two members of the phytochrome family are regulated and can initiate signaling responses to different light cues despite highly similar absorption spectra:

- phyA can initiate downstream responses as a PfrA–PrA heterodimer, while for phyB the active species is PfrB–PfrB^3^. Given that there is no cooperativity for the Pr to Pfr conversion this partly explains why phyA can trigger responses with a much lower fraction of Pfr. Moreover, the requirement for PfrB homodimers leads to a better differentiation of the phyB and phyA action spectra, with a sharper decline of phyB responses at longer wavelength^3^.
- phyA nuclear import by the FHY1/FHL nucleo-cytoplasmic shuttling protein allows phyA to enter the nucleus where it triggers essentially all known responses even with very low levels of PfrA. In the nucleus phyA releases FHY1/FHL presumably as it returns to PrA and undergoes a second light activation to interact with signaling elements (e.g. PIF, SPA). Hence efficient phyA signaling is favored in conditions promoting cycling between Pr and Pfr as in FR light. Moreover, in such conditions only a small fraction of phyA is ever activated and given that activated phyA is subsequently degraded this enables maintenance of a substantial pool of phyA. The shift in phyA action spectrum towards FR light can be explained to a large extent in a model including light-induced instability and the necessity for a double Pr–Pfr activation^27^.
- The ability of phyA to interact with signaling partners such as PIF1 and PIF3 in conditions (e.g. continuous FR) where phyB cannot may further explain the different response modes.

The presence of both modes of phytochrome signaling enables angiosperms to respond to a broader range of light environments. It was proposed that increasing vegetation cover on land, which led to a greater variety of light environments, favored the emergence and maintenance of both modes of action^2^. However, phyA and phyB action also lead to antagonistic responses in some conditions. For example, under a plant canopy low R/FR inhibits phyB, which releases stem growth inhibition while it activates phyA, which inhibits elongation. Hence, the activity of both photoreceptors has to be controlled in space and time. Regulated abundance contributes to this with phyA being 8.5 times more abundant than phyB in dark-grown seedlings allowing the transition to photo-autotrophic life even in the shade of competitors^26^. In light-grown seedlings phyB becomes the most abundant phytochrome allowing seedlings to respond to shade cues (low R/FR) by promoting stem elongation to reach unfiltered light.

## Structure of phytochrome molecules and link to function

**Fig. 2:**
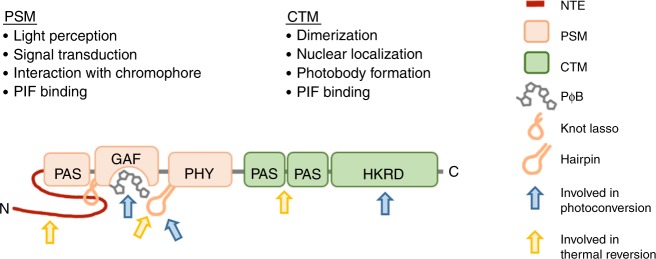
Structural domains of phytochromes and their role in perception of environmental signals and downstream signaling. NTE N-terminal extension, PSM photosensory module, CTM C-terminal module. Modified from Burgie et al.^44^

Plant phytochromes are dimeric, each monomer consisting of a ~1150 aminoacids covalently bound to its chromophore, a linear tetrapyrrole named phytochromobilin (PΦB) (Fig. 2) (for a comprehensive review on phytochrome structures we suggest Burgie et al.^1^). Crystal structures of the phyB N-terminal photosensory module (PSM), solid-state NMR analysis of Oat phyA PSM in the Pr and Pfr form, and structures of bacterial and cyanobacterial phytochromes are available and inform our mechanistic understanding of plant phytochrome structure and how it pertains to subsequent signaling steps^33–35^.

The apoprotein can be divided in the N-terminal PSM, which consists of the N-terminal extension (NTE), for which structural information remains scarce, and three structurally related domains Period/Arnt/SIM (PAS), cGMP phosphodiesterase/adenylyl cyclase/FhlA (GAF), and a phytochrome-specific domain (PHY) and a C-terminal module (CTM) comprising two PAS domains and a histidine kinase-related domain (HKRD)^1^ (Fig. 2). In the following paragraphs we will describe each part of the protein and its role in light-regulated protein interactions.

The chromophore is bound covalently to a conserved cysteine in the GAF domain, which has intrinsic chromophore lyase activity^1,35^. Light perception triggers a Z to E isomerization around the C15–C16 double bond of PΦB, which leads to a cascade of structural modifications in the protein^1,34,35^. PΦB interacts with the GAF domain covalently, but also has non-covalent interactions with residues from the NTE, PHY, and PAS domains. Hence all domains of the PSM are affected by the light-induced conformational change of the chromophore^1,35^. In particular, the hairpin (or tongue) structure in the PHY domain undergoes a beta-sheet to alpha-helix transition resulting in broader structural rearrangements^1,35,36^. This affects the figure-of-eight knot present between the PAS and GAF domains pulling them together, while separating the PHY domains from each monomer^1,34,35^. Initial conformational rearrangements in the PSM lead to structural changes also affecting the CTM. Ultimately, the light-induced Pfr conformer interacts with other proteins necessary for nuclear localization and signaling partners such as transcription factors of the PHYTOCHROME INTERACTING FACTOR (PIF) family and ubiquitin E3 ligase complexes^37–42^.

The NTE is longer in plant than bacterial phytochromes and variations in its sequence explains some differences between type I and type II phytochromes. In particular, type II phytochromes have a longer NTE, and in Arabidopsis replacing phyB NTE-PAS with that of phyA confers two features to this hybrid phytochrome that are unique to phyA; the capacity to accumulate in the nucleus in response to FR and rapid degradation in R^43^. This part of the protein is also involved in the control of thermal relaxation^1,44^ (Fig. 2). Both in phyA and phyB phosphorylation of the NTE modulates this reaction^45–49^. Several residues from the GAF and PHY domains control Pfr stability, thermal relaxation, and hence the ability of phyB to respond to light and temperature cues^1,5,44^. Finally, the PHY domain comprises key determinants distinguishing phyA from phyB, which may relate to different rates of thermal relaxation^20,43^. In summary, despite strong conservation several domains of the PSM contribute to subfunctionalisation of type I and type II phytochromes.

The presence of the HKRD suggested that phytochromes transduce their signal through the CTM. Although many bacterial and cyanobacterial phytochromes have a C-terminal histidine kinase domain and act as light-regulated histidine kinases, plant phytochromes are not histidine kinases and their role as Ser/Thr kinases remains contentious^50,51^. Actually, the PSM fused to a nuclear localization signal and a dimerization sequence is sufficient to restore most phyB functions, pointing to key signaling functions of the PSM^52^. Major roles of the plant phytochrome CTM are dimerization, nuclear import, and localization to sub-nuclear structures known as photobodies^44,52,53^. However, it was recently shown that the C-terminal part of phyB also engages in light-regulated interactions and regulation of PIF activity^39,40^. Moreover, the activity of the CTM is controlled by post-translational modification with SUMOylation limiting the ability of active phyB to interact with downstream signaling targets thereby limiting light responses^54^. In addition, the CTM modulates active (Pfr) phytochrome levels with the HKRD inhibiting the Pr–Pfr photoconversion while the PAS–PAS promotes thermal reversion^44^ (Fig. 2). Hence, while the division of plant phytochromes into PSM and a CTM helps describing the molecule, both parts of the photoreceptor contribute to the regulation of active Pfr levels and downstream signaling activities.

## Light-controlled nuclear import of phytochromes

Upon light perception all five phytochromes in Arabidopsis are translocated from the cytosol to the nucleus. This is a key step that is required for almost all known phytochrome responses^55–58^, it is broadly conserved in all land plants studied and also in marine algae^57,59,60^. The molecular mechanisms as well as the dynamics of this transport are distinct for type I (phyA) and type II (mostly studied for phyB) phytochromes, which contributes to the unique signaling features of phyA^27,57^ (Box 1). While phyA nuclear import relies on interaction with NLS-containing proteins, how phyB enters the nucleus remains poorly understood^57^.

phyA enters the nucleus very quickly even in conditions leading to very low levels of phyA in the active conformation (PfrA) (VLFR and FR-HIR), hence the heterodimer PfrA–PrA is probably transported to the nucleus^57^. This translocation depends on the interaction between PfrA and the NLS-containing proteins FHY1 and FHL^56^. The NLS in FHY1 is recognized by importin α, allowing nuclear import of the complex^61^. According to modeling approaches, once in the nucleus phyA is released from FHY1 and has to undergo another cycle of photo-activation to initiate light responses^27^. FHY1 exits the nucleus through the exportin route, and its import or export depend on its phosphorylation state^61^. FHY1 is rapidly phosphorylated in R light thereby limiting phyA nuclear localization and providing an attenuation mechanism for phyA responses to R^62^. This import mechanism has deep evolutionary roots as an FHY1-like protein is present and required for phytochrome responses in the basal land plant *Marchantia polymorpha*^63^ and FHY1-mediated phytochrome nuclear import operates in the moss *Physcomitrella patens*^60^.

Currently there is no consensus about the molecular mechanisms for phyB–E nuclear localization^57^. phyB nuclear import is induced by the same signals capable to induce the LFR and both PfrB–PfrB and PfrB–PrB dimers can enter the nucleus^3^. It was proposed that phyB nuclear import is mediated by light-induced NLS-unmasking, but no canonical NLS sequence is recognizable in phyB^53^. phyB import is not controlled by FHY1 or FHL, but may occur following interaction with NLS-bearing PIF transcription factors^64^. Although less studied some information about phyC–E localization is available in Table 1. While the mechanisms of type II phytochromes nuclear import remain poorly understood, it is clear that the distinct modes of type I versus type II phytochrome nuclear import are an important feature underlying photoreceptor subfunctionalization.

## Phytochrome signaling elicited through light-activated interactions

**Fig. 3:**
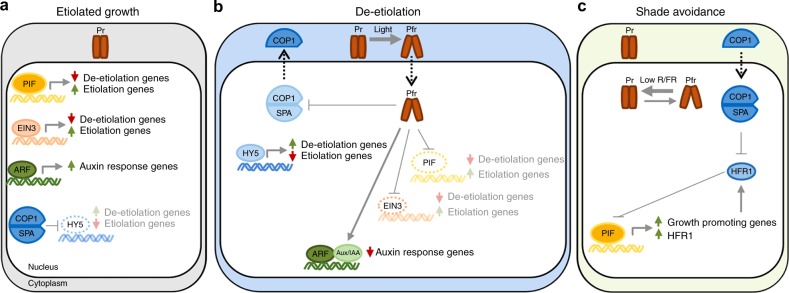
Simplified mechanism for phytochrome control of transcription factors in different light environments. **a** Below the soil surface during etiolated growth. For simplicity we consider that phytochromes remain inactive (Pr) below the soil surface, which results in accumulation of transcription factors PIFs, EIN3, and ARFs and subsequent induction of etiolation and auxin response genes. The COP1/SPA ubiquitin E3 ligase accumulates in dark and leads to proteasome-mediated degradation of HY5, a transcription factor that suppresses the expression of genes required for etiolation and induces expression of genes required for de-etiolation. **b** During de-etiolation upon light perception. Light perception activates phytochromes (Pfr) which promote de-etiolation by directly inhibiting PIFs and EIN3, and indirectly inhibiting ARFs by stabilizing Aux/IAA proteins. The Pfr form of either phyA or phyB interacts with SPA proteins, resulting in inhibition of COP1/SPA. This results in stabilization of HY5 leading to induction of de-etiolation related gene expression and repression of etiolation genes. **c** In a deetiolated plant in response to shade (reduced R/FR). Low R/FR in shade reduces the fraction of active phytochrome (Pfr/Ptot). PIFs accumulate and induce growth-promoting gene expression. In addition, PIFs induce a negative feedback loop exemplified by *HFR1* expression. HFR1 (and other HLH proteins) binds to PIFs forming non-DNA-binding heterodimers. COP1/SPA is also involved in this loop by leading HFR1 to proteasome-mediated degradation. Arrows indicate positive regulation, blunt-ended arrows indicate negative regulation, and dotted-lined arrows indicate nucleo-cytoplasmatic relocalization

**Fig. 4:**
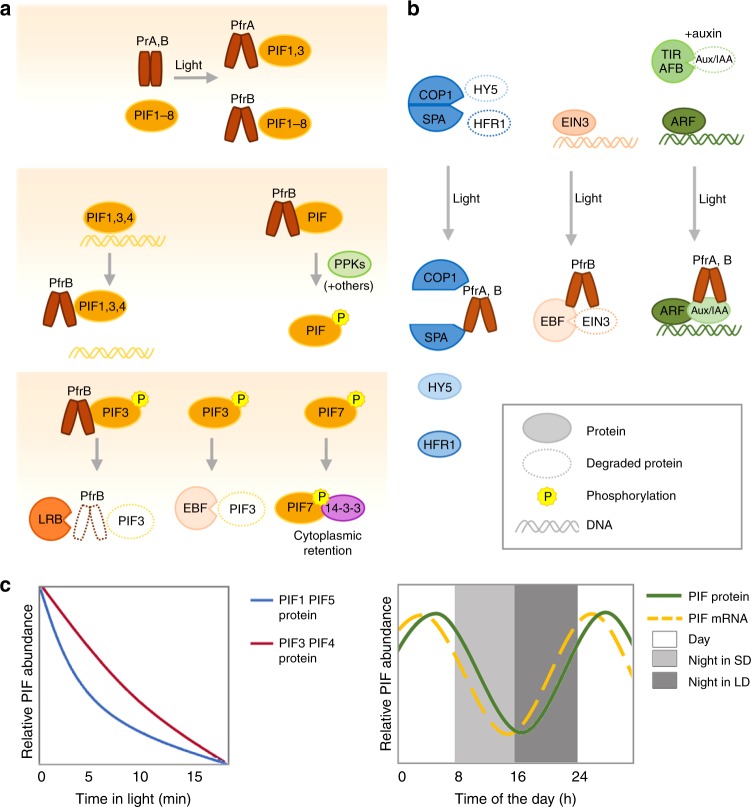
Mechanisms of phytochrome-mediated regulation of transcription factors. **a** From top to bottom, sequential steps by which Pfr inhibits PIFs. Top: PfrA interacts with PIF1 and PIF3 while PfrB interacts with PIF1–PIF8. Middle left: for PIF1, 3 and 4 phytochrome inhibits DNA binding. Middle right: Interaction with Pfr leads to phosphorylation of PIFs. Many kinases have been found to phosphorylate PIFs (see text) with PPKs phosphorylating PIFs in response to light. Bottom: after light-induced phosphorylation, PIF3 is degraded by LRBs and EBFs with phyB co-degradation occurring in the LRB-mediated process (left, center), phosphorylated PIF7 interacts with 14-3-3 proteins and remains in the cytoplasm (right). **b** Other mechanisms of transcriptional control by phytochromes. Left: PfrA and PfrB interact with SPA and inhibit the COP1/SPA complex. Center: PfrB interacts with EIN3 to promote ERF-mediated EIN3 degradation. Right: PfrA and PfrB interact with Aux/IAA to prevent their degradation by SCF^TIR1/AFB^. **c** Patterns of PIF abundance depending on the developmental state and growth conditions. In etiolated seedlings PIFs accumulate to high levels, promoting etiolated growth. Upon light exposure, PIFs are rapidly degraded in a phytochrome-dependent manner, with half-lives of ~5 min for PIF1 and PIF5, and ~10 min for PIF3 and PIF4 (left). In contrast, in light-grown seedlings PIFs are under strong transcriptional control, allowing accumulation of the protein even in conditions when phytochrome activity is predicted to be high (right), SD (short days), LD (long days)

The light-induced Pfr conformer selectively interacts with several classes of transcription factors and with ubiquitin E3 ligases, which control the stability of transcriptional regulators. Collectively these light-regulated interactions result in rapid and global transcriptional reprograming^41,58,65,66^. We will start by describing the consequences of phytochrome–transcription factor interactions (Fig. 3) before a brief presentation of how phytochromes regulate ubiquitin E3 ligases (e.g. COP1/SPA, DDB1/DET1) and convergence points between both signaling branches (Figs. 3 and 4).

PIFs are members the basic helix–loop–helix (bHLH) TF-superfamily that selectively interact with light-activated phytochromes^41,65,67^. Pfr interacts with PIFs through short domains located towards their amino-terminus. These short sequences are known as APB for Active PhyB binding and APA for Active PhyA binding domains. Arabidopsis has eight PIFs (PIF1, PIF2/PIL1, PIF3-PIF8) with an APB motif, whereas an APA motif is only present in PIF1 and PIF3 (refs. ^41,65^) (Fig. 4a, upper panel). The phy-PIF signaling module originated early in evolution since the liverwort *Marchantia polymorpha* has a single phytochrome and a single APA-containing PIF^68^. PIF–Pfr interaction results in PIF inactivation, which occurs through several mechanisms^41,65,68,69^ (Fig. 4a). First, phyB–PIF interaction results in blocking the DNA-binding capacity of several PIFs (shown for PIF1, PIF3, PIF4 while this was not reported for other PIFs)^39,40,70^. Second, interaction with the photoreceptor leads to rapid PIF phosphorylation which for most PIFs is followed by ubiquitination and proteasome-mediated degradation. PIF7 is an exception in this regard as phosphorylation facilitates its interaction with 14-3-3 proteins leading to cytoplasmic retention of the transcription factor^41,65,71^ (Fig. 4a). Moreover, PIFs are under strong transcriptional control^72^. This allows PIF accumulation during the day, even when phytochromes are active^18,73^ (Fig. 4c, right panel). In summary, light-activated phytochromes inhibit PIFs through multiple mechanisms, which presumably contribute to optimal light regulation of these transcription factors across a variety of light conditions^40^ (Fig. 4a, c).

The phytochrome-PIF regulon is an initial step underlying photoreceptor’s control of a wide array of physiological and developmental responses^18,19,41,58,65^ (Figs. 3 and 4). However, it was later discovered that PIFs regulate additional processes in response to other external and internal cues acting as signaling hubs^41,65^. This may explain why PIF phosphorylation and subsequent proteasome-mediated degradation is so complex^41^. As mentioned above, plant phytochromes contain a C-terminal HKRD but surprisingly a recent report concluded that the PSM of phytochromes (including phyB) directly phosphorylates PIFs^50^. However, phyB-mediated PIF3 phosphorylation was not observed in a subsequent study and based on the phyB PSM structure it is unclear how it may act as a protein kinase^51^. Moreover, while both the PSM and CTM of phyB bind and inhibit PIF3, it is binding to the CTM which results in PIF3 degradation, which typically follows PIF phosphorylation^38,39,74^. Members of Photoregulatory Protein Kinases (PPK1–4; formerly called MUT9-Like Kinases (MLKs)) are the best-characterized kinases phosphorylating PIF3 in a light-induced fashion^41,51^ (Fig. 4a). These protein kinases are recruited to the nuclear PfrB–PIF3 complex leading to PIF3 phosphorylation and subsequent degradation^51^. This study reports a large overlap between PIF3 phosphorylation sites determined in vivo and following in vitro phosphorylation by PPK1. Interestingly, phyB enhances the protein kinase activity of PPKs towards PIF3 thereby potentially acting as a pseudokinase rather than a genuine protein kinase^51^. While the role of PPKs in PIF3 phosphorylation and subsequent degradation is supported by mutant analysis, the growth phenotype of *ppk* mutants is somewhat counter-intuitive. This might be due to a broader role of these protein kinases including light-induced phosphorylation and subsequent degradation of phyB and cryptochrome 2 (refs. ^51,75^). However, precisely delimiting PPK function in phytochrome signaling is hampered by the essential nature of the *PPK* gene family^51^. Additional protein kinases have been implicated in PIF phosphorylation and subsequent proteasome-mediated degradation including BIN2, CK2, and MPK6 (refs. ^73,76–78^) (reviewed in ref. ^41^). BIN2 phosphorylates and regulates the stability of PIF4. However, this does not specifically pertain to phytochrome-mediated PIF regulation but rather to the PIFs-brassinosteroid signaling crosstalk, which is important for plant growth regulation^65,73,79,80^.

PIF ubiquitination and subsequent proteasome-mediated degradation is equally complex involving several CULLIN (CUL) RING UBIQUITIN LIGASEs (CRLs)^74,81–85^. Five different families of substrate recognition components have been implicated in the ubiquitination of different PIFs through three members of CRLs: CUL1, CUL3, and CUL4. PIF1 is ubiquitinated by CUL4 ^COP1/SPA^ and CUL1^CTG10^; PIF3 by CUL3^LRB1/2/3^ and CUL1^EBF1/2^; PIF4 by CUL3^BOP1/2^; and PIF5 by CUL4^COP1/SPA^^74,81–85^. LRB1-3 and EBF1/2 control light and phytochrome-dependent ubiquitination and subsequent degradation of PIF3 (Fig. 4a, lower panel). Interestingly, LRBs are preferentially involved in strong-light-induced PIF3 degradation in a process resulting in PIF3-phyB co-degradation, while EBFs are more important in response to low light and target PIF3 but not phyB^74,81^. Moreover, light-regulated PIF3 abundance is controlled transcriptionally by EIN3, which is itself destabilized by light through phyB-promoted interaction with CUL1^EBF1/2^ (ref. ^86^) (Fig. 4b, middle panel). The role of other E3 ligases in regulating PIF abundance is not as clearly light and phytochrome-regulated. COP1/SPA’s role is complex because the most striking feature of *cop1* mutants is the reduction of PIF abundance in the dark, suggesting that this E3 ligase stabilizes PIFs in darkness^41,84,87^. COP1 controls PIF3 levels in etiolated seedlings indirectly through BIN2 kinase inhibition, thereby limiting BIN2-mediated PIF3 destabilization^73,77^. COP1 was also implicated in the light-induced degradation of the light-labile phyA. However, subsequent studies showed that when grown in more natural conditions (on soil) phyA degradation occurs normally in *cop1* mutants demonstrating the difficulty of interpreting the rather pleiotropic *cop1* phenotype^88^. BOPs control PIF4 ubiquitination and subsequent degradation but although the process is more efficient in the light it is not strictly light dependent and also contributes to the regulation of PIF4 abundance in darkness^82^. Finally, CTG10 was recently shown to control light-modulated PIF1 abundance in seeds and seedlings but the underlying mechanism remains to be established^85^. The involvement of multiple PIF degradation systems is intriguing and may suggest specific mechanisms dedicated to particular PIFs, cell types, organ, and/or stimuli regulating PIF abundance.

More recently, direct phytochrome control of transcription factors was extended to additional families. This includes phyB-EIN3 interaction, which facilitates EIN3-SCF^EBF1/2^ complex formation, EIN3 ubiquitination, and degradation^86^ (Figs. 3b and 4b, middle panel). There is some evidence for inhibition of the bHLH protein BES1, a central player in brassinosteroid signaling, following interaction with phyB^89^. Finally, several Aux/IAA regulators of auxin-controlled gene expression are modulated through interaction with phyA or phyB^90,91^ (Figs. 3b and 4b, right panel). In these last examples, Aux/IAAs were protected from SCF^TIR/AFB^ auxin-regulated proteolytic degradation following interaction with the phytochromes^90,91^. Phytochrome-regulated PIF activity regulates plant hormone signaling by directly targeting the expression of key hormone biosynthetic and signaling genes^17,66,92–96^. This enables light-regulated hormonal responses which in some cases feedback on PIF activity. For example, phytochrome regulation of gibberellic acid (GA) levels controls the stability of the DELLAs family of growth-suppressing proteins^97^. Light-independent interaction between DELLAs and PIF1, PIF3, PIF4, and PIF5 leads to proteasome-mediated PIF degradation independently of phyB and LRBs^98^. Upon interaction with DELLAs PIF3 and PIF4 are also prevented from binding to DNA^99,100^. These mechanisms contribute to an important and complex crosstalk between light and hormonal-mediated growth and developmental responses^17,41,60,65^.

Phytochromes also exert a strong effect on gene expression through light-activated inhibition of ubiquitin E3 ligases in particular COP1/SPA and DDB1/DET1 (Figs. 3 and 4b, left panel)^42,58^. Inhibition of COP1/SPA is initiated by selective interaction of the Pfr form of either phyA or phyB with SPA proteins^101,102^. The mechanisms underlying photoreceptor-mediated inhibition of COP1/SPA and the main targets of this regulation are described in recent reviews^42^. COP1 and SPA proteins are present in green algae and all land plants but the central role of COP1/SPA in light signaling was proposed to originate in the ancestor of angiosperms^103,104^. We will briefly illustrate the role of COP1/SPA in phytochrome signaling and links between COP1/SPA and PIF-regulated transcription. The bZIP transcription factor ELONGATED HYPOCOTYL 5 (HY5) is a prominent target of this regulation. HY5 is stabilized following light stimulation and is an important regulator of light-controlled development (Fig. 3b). Interestingly, HY5 and PIFs share many target genes but control them in antagonistic fashion. This system allows for repression of photosynthetic genes in darkness followed by their light activation by a light-controlled exchange of major transcription factors^105,106^. Some members of B-box (BBX) protein zinc-finger transcription factors, including CONSTANS, a major determinant of photoperiod-induced flowering, are controlled by COP1 and modulate numerous aspects of photomorphogenesis^42,107–109^. Finally, several transcriptional regulators targeted by COP1 directly inhibit PIFs, for example, by forming non-DNA-binding heterodimers (e.g. HFR1). As PIFs directly control *HFR1* expression this constitutes a negative feedback loop with important growth regulatory properties operating, for example, during the shade-avoidance response^15,110,111^ (Fig. 3c). This final example also illustrates a salient feature of phytochrome-regulated gene expression with the photoreceptor directly regulating multiple steps of a gene regulatory network^41,42,58^.

## New modes of action of phytochromes

In addition to regulating a number of transcription factors, phytochromes regulate other aspects of transcription, post-transcriptional regulation, and translation. A genome-wide study indicates that phytochromes potentially regulate alternative promoter selection of more than 2000 genes^112^. This frequently results in modified protein amino-termini with predicted alteration of subcellular localization in several hundred cases^112^. Of particular interest is an essential photorespiration enzyme, glycerate kinase (GLYK) which changes its location from chloroplasts to the cytoplasm in shade-treated plants, thereby alleviating photoinhibition occurring in fluctuating light conditions^112^. ChIP data indicate binding of phyA and phyB to numerous promoters and phyB-binding sites are significantly enriched in genes showing phytochrome-mediated alternative promoter usage^4,112,113^. How phytochromes associate with chromatin, the functional consequence of this interaction and the mechanisms underlying phytochrome-mediated alternative promoter usage remain to be determined. Post-transcriptionally, phytochromes may control alternative splicing (AS) (extensively reviewed in Cheng et al.^114^). In *Physcomitrella patens* phytochromes appear to directly regulate AS^115^. In Arabidopsis, despite the identification of a number of splicing factors that can interact with phytochromes^116,117^, it remains debated whether AS is due to direct phytochrome regulation, is an indirect metabolic consequence of de-etiolation or a combination of both^114,118^. Light also regulates AS through chloroplast retrograde signaling, a process requiring normal chloroplasts^119^. Considering the recent discovery of a second-site mutation in the *VENOSA4* gene controlling chloroplast size and photosynthetic traits in the frequently used *phyB*-9 allele (used in aforementioned studies)^120^, we conclude that the extent to which phytochromes rely on AS to control development requires further investigations.

De-etiolation is accompanied by extensive changes not only in transcript abundance and splicing but also translation. In etiolated seedlings hundreds of transcripts are present in cytosolic processing bodies (p-bodies) in a translationally repressed state^121^. Light cues, in a phytochrome-dependent manner, inhibit p-body formation/maintenance allowing translation of those mRNAs. Light-regulated suppression of translational inhibition is functionally important for seedling de-etiolation^121^. However, how phytochromes control this step remains poorly understood with the exception of light-induced inhibition of protochlorophyllide reductase A (*PORA*) translation that is mediated by a PENTA (PNT1)–PfrA complex^122^. Whether phytochromes are more broadly involved in translational control awaits further investigation. However, this may be a more downstream phytochrome signaling effect given that most tested physiological responses require nuclear rather than cytosolic phytochrome^57^.

## The role of photobodies

Once in the nucleus all phytochromes locate in nuclear foci termed photobodies (PBs)^57,58,60,123^. During de-etiolation, a set of small PBs containing phyA and phyB appear after 2 min of irradiation, they disappear after 1 h and a new set mainly containing phyB is established after 2 h^58,87^. The abundance and size distribution of these late PBs can subsequently be modified by light conditions that change the fraction of active phyB. In de-etiolating seedlings, increasing R intensity or the R/FR ratio changes phyB localization from small PBs to increasingly large PBs^58,123^. Moreover, in adult plants phyB PBs are modulated by shade cues with low R/FR or low irradiance rapidly converting big PBs to many small PBs^124^. The same reduction in PB size is observed when light-grown seedlings are transferred into the dark^125^. Although light has a dominant effect, phyB PBs size distribution is also affected by ambient temperature^5^. Association with PBs largely depends on the C-terminus of phyB^123^. Moreover, phyB mutants with faster thermal reversion have smaller PBs^126^ while a constitutively active phyB forms PBs in the dark^127^. Finally, other proteins regulate the size and stability PBs. HEMERA (HMR), NUCLEAR CONTROL OF PEP ACTIVITY (NCP) and REGULATOR OF CHLOROPLAST BIOGENESIS (RCB), three dual targeted nuclear/chloroplast proteins are necessary for large PBs formation^128–130^. In addition, PHOTOPERIODIC CONTROL OF HYPOCOTYL 1 (PCH1) and its paralog PCH1-LIKE (PCHL) stabilize these sub-nuclear structures^131–133^. Recent reviews provide a more comprehensive list of PB functions and PB-associated proteins^57,58^.

Despite the tight correlation between phytochrome sub-nuclear localization and function, it is still not clear which molecular processes occur in each type of PBs. Early PBs have been proposed as the site of PIF degradation, since phyB PBs depend on PIF3 (ref. ^87^) and the abundance of those PBs correlates with PIF3 degradation^58,128^ (Fig. 4c, left panel). Late PBs are considered as sites maintaining active phyB^3,131–133^. PCH1 and PCHL are structural components of PBs which interact with phyB^131–133^. PCH1–phyB interaction inhibits Pfr thermal reversion in vitro^133^, while both PCH1 and PCHL reduce phyB thermal reversion in vivo, thereby restricting hypocotyl growth during the night period in short days and at elevated temperature^131–133^. Interestingly, PCH1 and PCHL expression is circadian regulated peaking at dusk correlating with large phyB PBs and possibly contributing to the diel regulation of PB formation^131,134^. While these results support the notion that PBs are a site for storage and maintenance of phyB Pfr, the presence of other proteins in these late large PBs points to additional functions. COP1 and SPA proteins co-localize with phytochromes in PBs. Originally this was interpreted as a possible role for PBs in phytochrome degradation. However recent work showed that the interaction of phytochromes with SPA proteins in PBs inactivates the COP1–SPA complex, allowing the accumulation of their targets, supporting the idea that PBs are site of phytochrome signal transduction^101^ (Figs. 3 and 4b). Additional proteins co-localize with PBs and support a role of PBs in phytochrome signaling. TZP, a chromatin-binding protein containing a zinc-finger domain and a PLUS3 domain, interacts with both phyA and phyB in a light-dependent manner and co-localizes to PBs consistent with a role of PBs in phytochrome-regulated transcriptional regulation^135,136^. SFPS interacts with PfrB and co-localizes with phyB and U2 small ribonucleoprotein (U2 snRNP)-associated proteins in nuclear bodies, suggesting that PBs could be a site of storage or modification of splicing factors and could have a role in the regulation of mRNA post-transcriptional modifications in response to light signals^117^. Finally, HMR, NCP, and RCB control PB size and are closely connected to PIF-mediated phytochrome signaling. All three proteins are required for light-induced degradation of PIF1 and PIF3 (refs.^128–130^). Moreover, HMR may also act as a transcriptional co-activator working with PIFs suggesting that at least in some cases PIF transactivation and stability are intimately linked^58,137,138^. Finally, HMR, NCP, and RCB control expression of chloroplastic gene expression in a process linking PIF degradation with activation of the plastidic PEP RNA polymerase^129,130^. These studies reveal how early phytochrome-PIF signaling steps lead to coordinated regulation of nuclear and chloroplastic gene expression which is particularly important for the transition towards photo-autotrophy.

## Phytochrome signaling from the cellular to the whole-plant level

While we started by describing phytochrome signaling at the cellular level, not all phytochrome-mediated responses are local. Examples where the site of light perception is far from the physiological or developmental response are numerous and have been reviewed recently^17,139,140^. Many phytochrome-triggered long-distance signals rely on hormones. Phytochromes through PIF regulation control the expression of genes encoding rate-limiting catalytic enzymes of several phytohormones (e.g. auxin, gibberellins, abscisic acid), presumably at the site of light perception^17^. Regulated transport and response to these hormones underlies some of the distal responses elicited by phytochromes^141^. In other cases small proteins like HY5 or FLOWERING LOCUS T (FT) travel long distances to trigger a phytochrome-dependent response^9,142^. However, in many cases the long-distance signal remains to be established^139,143^. In the following paragraphs we will describe the most recent examples of phytochrome-mediated local and long-distance responses throughout development by starting with germination and finishing with the transition to reproduction.

In seeds, shortly after imbibition shade signals can inactivate phyB in the endosperm overriding phyA-dependent signaling in the embryo and preventing germination. This is achieved by controlling the gibberellin to abscisic acid balance and abscisic acid transport from the endosperm to the embryo^17,144^. Etiolated seedlings have a strong gravitropic response that depends on amyloplasts in the endodermis. It was recently shown that phyB-mediated light perception inhibits gravitropism by inhibiting PIFs, which allows the conversion of gravity-sensing amyloplast to chloroplast-like plastids in the endodermis. Unexpectedly this response requires phyB expression in the epidermis while PIF1 has to be present in the endodermis^145^. Moreover, PIF1 degradation in the endodermis occurs very efficiently even when phyB is selectively expressed in the epidermis^145^. This is surprising because all known mechanisms for phytochrome-mediated inhibition of PIFs depend on interaction of both proteins and it suggests that an unknown signal travels very fast from the epidermis to the endodermis.

After de-etiolation, shade signals promote hypocotyl growth while reducing cotyledon expansion^8,95,146,147^. This response largely depends on inactivation of phyB in the cotyledons which allows PIFs to promote the expression of *YUCCA* genes, coding for rate-limiting auxin synthesis enzymes. Auxin is then transported to the hypocotyl where it promotes elongation^17,139,147^. Shade signals also increase carbon allocation from cotyledons to hypocotyl by sucrose transport, which may contribute to the contrasting growth responses between cotyledons and hypocotyls^11^. In addition, local auxin production also contributes to hypocotyl elongation, but whether this relies on local shade perception or on another distal cotyledon-derived signal remains to be established^148^.

In adult plants shade signals can be more complex, for instance they might be caused by self-shading instead of shading by competitors. Consequently, shade responses are also more complex often involving interorgan signaling. Low R/FR perceived by phyB at the leaf tip promotes leaf hyponasty. Similar to young seedlings, the long-distance signaling relies on auxin synthesis promoted by PIF-mediated *YUCCA* expression in the blade and auxin transport towards the base of the petiole^149,150^. In contrast, a local drop in R/FR on the petiole does not promote hyponasty but instead promotes petiole elongation^149,150^. Gene expression analyses combined with physiological studies indicate that auxin biosynthesis and response are regulated differently in different organs and are both indispensable for robust shade-avoidance responses^80,95,110,146,149–151^. This includes a predominant function of PIF7 in the regulation of auxin biosynthesis genes while PIF4 and PIF5 also regulate auxin signaling genes^80,93,94,96,110^. Interestingly, in some cases PIF4 works as a member of tripartite TF-complex with AUXIN RESPONSE FACTOR 6 (ARF6) and BRASSINAZOLE RESISTANT 1 (BZR)1^79^. Moreover, phyA and phyB potentially directly stabilize Aux/IAAs in the light^90,91^ (Figs. 3 and 4), suggesting another level of local regulation of auxin signaling by phytochromes. Under persistent shade new leaves develop less stomata in a phyB-dependent manner. This is independent of phyB expression in the stomata lineage, and phyB can regulate stomata development in young leaves depending on the light conditions perceived in older leaves^152^. In this case phyB activity controls the expression of genes related to stomata development as SPEECHLES and MUTE but the mobile signal has not yet been identified^152^.

In addition to a role in aerial organs shade signals also control root architecture. This phenomenon can be explained by two alternative hypotheses. Either phytochromes in the shoot perceive light and send a chemical signal to the roots, or light reaching the shoot is piped towards the root where it is perceived by root-localized phytochrome triggering a local response. So far there is evidence supporting both theories^9,10^. In both cases, the phytochrome downstream signaling molecule involved is HY5. This protein is known to travel long distances through the phloem^153^, it is stabilized in the shoot in response to light and is translocated to the root to control root architecture^9^. However, according to grafting experiments a full response requires a functional HY5 gene also in the root^10^. One possible explanation is that HY5 promotes its own expression^154^, hence shoot derived HY5 may promote HY5 expression in the root, but further studies are needed to address this model.

A final example of long distance signaling by a small protein is the case of flowering induction by FT. Light perceived by phytochrome in the leaves control FT abundance in response to photoperiod and shade signals. Upon the perception of the inductive signals FT accumulates in leaves and travels to the shoot apical meristem where it induces differentiation of the reproductive structures^17,142^. Thus, although phytochromes are expressed in all plant tissues and the early steps driving activation are conserved, phytochrome responses in the different organs presumably result from local, distal, or a combination of both types of signals.

## Phytochrome signaling in other flowering plants

Many of the molecular mechanisms underlying phytochrome signaling have been discovered in Arabidopsis. However, phytochrome research has a long history and early studies have used a variety of plants leading to the discovery of these photosensory receptors and their functions^155,156^. Based on an ever-growing list of sequenced genomes we now know that many components of phytochrome signaling are deeply conserved in land plants^103^. Moreover, in a number of angiosperms including crops, genetic analyses identified light responses controlled by phytochrome signaling components^20,157,158^. In this section we will mention similarities and differences between Arabidopsis and other species in terms of phytochrome-mediated responses.

Most phytochrome responses described in Arabidopsis are shared by other plant species. This includes regulation of germination, de-etiolation, responses to shade and photoperiodic control of flowering time^20,155,157,158^. All angiosperms have phyA and phyB class phytochromes with variable numbers of family members in each class^20^. Functional divergence between both classes is conserved with, for example, phyA controlling the FR-HIR and phyB mediating reversible LFR responses^20^. Most angiosperms also have a phyC class, which was proposed to have a more prominent role in grasses and possibly have a different mode of action than Arabidopsis phyC^159,160^. However, both in grasses and Arabidopsis phyB plays a broader role than phyC while phyC plays a particularly important role for photoperiodic regulation of flowering presumably as a phyB–phyC heterodimer^14,23,160,161^. We therefore conclude that additional research is required to conclude whether the mode of action of phyC differs among species.

Numerous flowering plants are shade avoiders but some species are shade tolerant and therefore display different adaptive responses to shade cues^155,162^. Phytochromes control both types of responses to foliar shade, and comparative studies between related species with contrasting shade responses suggest mechanisms underlying different adaptations to the same light cue^163,164^. In *Cardamine hirsuta*, a species related to Arabidopsis, shade tolerance can in part be explained by higher *PHYA* transcript levels and phyA protein activity^165^. In other comparisons between related species or varieties of *Geranium*, rice, or conifers, the differential responses to shade have been related to hormonal signaling^163,164,166^. The ecological significance of the shade-avoidance response has been demonstrated but in crops it may also produce undesirable consequences because it diverts resources from storage organs, decreases the ability of plants to defend against pathogens and makes them more vulnerable to wind damage^167^. In some species domestication led to reduced shade-avoidance responses presumably due to the selection of yield at high planting density^157^. The role of several phyB class phytochromes for shade responses is established in a number of crops including tomato, maize, wheat, and rice^161,168–171^. While some shade responses in crops trigger yield penalties, responding to shade cues is also beneficial^167^. A good example is the control of leaf positioning in dense stands of maize or sunflowers as shade signals promotes canopy gap filling and hence better usage of the light^172^. This may explain why temperate maize inbred lines maintain robust shade responses^168^.

A faster transition to flowering by shade cues is common in many crops, but in *Medicago sativa*, a perennial species, flowering is delayed by additional FR light^8,173^. This suggests that in perennial species, the strategy to survive in suboptimal light environments is to save nutrients for the next season rather than to trigger flowering as in annual species. In crop species flowering time is determinant for yield and phytochromes affect photoperiodic control of flowering in rice, sorghum, barley, soybean, wheat, among others^159,174–177^. Another photoperiodic response tightly linked to yield is tuberization. In potato, (*Solanum tuberosum*) reallocating resources towards tubers is controlled by a phyB and a phyC class phytochrome^178^.

Studies in a wide variety of species identified role for phytochromes in processes which are absent in Arabidopsis. For example, in tomato phyA, phyB1, and phyB2 control fruit nutritional quality, carotenoid synthesis, and time to ripening^179,180^. Interestingly, during fruit ripening the light signal to induce carotenoid biosynthesis originates in the fruit itself, bringing a new layer of complexity to the phytochrome system which cannot be studied in other models^179,180^. Phytochromes also control biotic interactions, which is well studied in the context of plant pathogen interactions, exemplified by shade cues which through inhibition of phyB also inhibit plant defense^167^. Such interactions can be complex as in tritrophic interactions where the plant attracts predators of the herbivore. A beautiful example of such interactions controlled by phytochromes was described in *Passiflora edulis* where shade signals inhibit the production of extra floral nectar^181^. Another example of phytochrome-controlled interaction with beneficial organisms comes from *Lotus japonicus* where phyB promotes nodulation^182^. This study is also interesting in the context of phytochrome-controlled nutrient uptake which is well established for photosynthesis but also comprises light regulation of nutrients uptake by roots^183^.

In most flowering plants signaling events downstream of phytochrome activation remain poorly understood. However, rapid and widespread phytochrome control of gene expression is conserved as exemplified by studies in rice and Arabidopsis^41,58,184^. Rice PIF transcription factors control many phytochrome-mediated responses including de-etiolation, chlorophyll abundance, senescence, gravitropism, elongation growth, and interestingly grain size which is of agronomic interest^41,185–187^. However, the role of HY5 in rice differs at least partially from its function in Arabidopsis as a rice *hy5* mutant is seedling lethal^188^. Characterization of *PIF* genes and mutants in maize shows that these bHLH transcription factors promote elongation of stem-like structures in etiolated seedlings and in response to shade as they do in Arabidopsis^41,189^. In tomato, PIFs control fruit ripening through a self-shading mechanism where light absorption by chlorophyll in the immature green fruit inhibits phytochrome activation^179^. At this stage low phytochrome activity allows PIF accumulation which inhibit carotenoid and tocopherol synthesis^179,190^. As fruits ripen, chlorophyll is degraded, which activates phytochromes triggering PIF degradation, thereby releasing PIFs inhibitory role on fruit maturation^179,190^. Regulation of tomato fruit quality involves a number of additional phytochrome signaling components including COP1, DET1, DDB1, and HY5 (ref. ^158^). Future studies will undoubtedly reveal how phytochrome signaling elements control light responses in a variety of species including agronomically important plants^41^.

## Outlook

Phytochromes are among the most studied proteins in the history of plant biology but despite many years of research, important questions remain unsolved. In the following paragraphs, we outline some such questions ranging from molecular to physiological and ecological scales.

At the molecular level the complete structure of a plant phytochrome remains unresolved. Also, we lack knowledge of how light regulates interactions between plant phytochromes and immediate downstream partners. Addressing these questions will require the use of rigorous biochemistry. For example, the affinity constants for complexes between light-activated phytochromes and PIFs or any other interacting partners (e.g. SPA, FHY1) have not been systematically studied. A rather substantial list of proteins that can interact with phytochromes exists. However, many such complexes have not been biochemicaly characterized to, for example, determine which complexes are the most likely to form or to learn about competition between different proteins interacting with phytochromes. Ideally, structures of phytochrome-PIF (or other interacting factor) complexes showing how light changes interaction surfaces will provide insights into these early light-triggered events. Moreover, given that phytochromes can interact with PIF proteins both through their PSM and CTM, such studies may reveal how these interactions lead to different modes of PIF inhibition. Biochemistry is also required to provide more insight into the mode of action of other proteins important in phytochrome signaling such as PCH1 and HMR. It should be noted that the common practice of assembling plant phytochromes with the cyanobacterial instead of the plant chromophore renders Pfr more thermally stable, which has obvious consequences on, e.g., the study of light-regulated interactions^44^.

At the subcellular level, while most phytochrome-mediated responses require nuclear localization there are some light-triggered responses which are too fast to be explained through phytochrome-mediated transcriptomic changes (reviewed in ref. ^191^). Cytosolic functions for phytochromes appear to be more prevalent in cryptogams, for example, in *Physcomitrella* there is evidence that phytochrome in the cytoplasm interacts with phototropins (which are located at the plasma membrane) to modulate phototropism^192^. However, in Arabidopsis phytochromes primarily control phototropism by controlling gene expression^140^. Once in the nucleus, understanding the function(s) of PBs remains an important challenge. Substantial evidence points to their importance as sites of PIF protein degradation, transcriptional control, potentially splicing and to maintain a pool of light-activated phytochromes. We understand how light-activated phytochrome links with transcriptional changes in particular via PIFs. However, what distinguishes different mechanism of phytochrome-mediated PIF inhibition, whether they mostly occur in different cells, organs, or developmental stages remains largely unknown. Finally, the links between different modes of phytochrome signaling are unclear. For example, does phytochrome-mediated alternative promoter usage depend on an initial transcriptional change or are these two independent modes of phytochrome action? Also, how do phytochromes regulate more long-term changes of the transcriptional landscape? Does this occur through epigenetic changes^96,193,194^, and how is this linked to early transcriptional changes?

Light and phytochromes affect numerous facets of plant development but for practical reasons only a limited number of phenotypes have been investigated in detail (e.g. seed germination, control of hypocotyl elongation). In some instances lessons learned from simple systems yield precious information to understand phytochrome responses in other organs. However, given that there is ample evidence for organ-specific phytochrome responses, focusing on other phenotypes such as leaf development will certainly yield interesting new insight. Such studies will also benefit from a better integration of cellular, developmental, and photo-biology. This includes understanding how phytochromes through the regulation of several classes of transcription factors regulate expression at the tissue and cellular levels. In addition, studying other species than Arabidopsis will broaden our knowledge of organ-specific phytochrome functions. In this sense, the improvement in CRISPR gene editing will be instrumental.

Despite the fundamental importance of using simple light conditions to understand phytochrome signaling^155,191^, experiments performed in more natural conditions may reveal important new roles of these photoreceptors and identify important factors controlling their activity. Most of our knowledge about Arabidopsis phytochromes is based on lab experiments with artificial light that does not have the same spectrum as sunlight and is typically about 10 times lower than what a plant would experience on a sunny day. In addition, light and temperature are mostly treated as binary parameter (on/off, high/low) without the gradual changes observed in nature. Moreover, strong and rapid fluctuations in light intensity that are common in nature are not captured in most experimental setups, with notable exceptions^195^. Using more realistic conditions led to new insights as exemplified by a study showing that in strong high R/FR phyA plays a role in de-etiolation, although phyA was typically regarded as not being important in high light^196^. Similarly, photoperiodic regulation of flowering time is quite different in standard lab conditions versus outside-grown plants^197^. This study suggests that the model explaining day-length-regulated flowering requires adjustments and shows that by better simulating natural light (R/FR) and temperature fluctuations data generated in the lab mimic outdoor experiments much better^197^. We hypothesize that in the natural context the role of other phytochromes (phyC, phyD, and phyE) and some phytochrome signaling elements may become more apparent. Moreover, using more realistic light environments is important to understand the crosstalk between phytochromes and other photosensory receptors.

In addition to more realistic environments more extensive use of genetic resources would be very informative. Although many of the phytochrome signaling elements are deeply conserved in land plants (e.g. phytochrome, PIF, COP1, SPA, HY5, and FHY1)^103^, there is substantial evidence for differences in light responses across Arabidopsis accessions that in some cases can be traced to the phytochrome or phytochrome-signaling elements^198,199^. Combined with the sequence of more than a 1000 accessions and increasingly detailed information about the collection site, this information is a fantastic resource to investigate adaptation to local light environments.
